# Tailored Surgical Treatment and Outcomes in Solid Pseudopapillary Neoplasms of the Pancreas: A Case Series of Five Consecutive Paradigmatic Cases

**DOI:** 10.3390/diseases14050180

**Published:** 2026-05-20

**Authors:** Arianna Pontrelli, Giovanna Di Meo, Francesco Paolo Prete, Piercarmine Panzera, Giuseppe Massimiliano De Luca, Natale Calomino, Maria Teresa Mita, Belinda De Simone, Michele Bisceglie, Monica Maria Miccoli, Alfio Gianalberto Testini, Michele Covelli, Massimo G. Viola, Luigi Marano, Mario Testini

**Affiliations:** 1 Department of Precision and Regenerative Medicine and Jonian Area (DiMePReJ), University of Bari “Aldo Moro”, 70124 Bari, Italy; arianna.pontrelli@policlinico.ba.it (A.P.); giovanna.dimeo@policlinico.ba.it (G.D.M.); piercarmine.panzera@policlinico.ba.it (P.P.); giuseppe.deluca@uniba.it (G.M.D.L.); michele.bisceglie@uniba.it (M.B.); monica.miccoli@uniba.it (M.M.M.); mario.testini@uniba.it (M.T.); 2 Department of Surgery, Di Venere Hospital, 70012 Bari, Italy; 3 Kidney Transplant Unit, Department of Medicine Surgery and Neuroscience, University Hospital of Siena, 53100 Siena, Italy; natale.calomino@unisi.it; 4 Department of Surgery, Cardinale G. Panico Hospital, 73039 Tricase, Italy; mt.mita@piafondazionepanico.it (M.T.M.);; 5 Department of General and Emergency Surgery, Bufalini Hospital AUSL Romagna Cesena, 47521 Cesena, Italy; 6 Department of Surgery, Sapienza University, 00161 Rome, Italy; 7 Department of Medicine, LUM University, 70010 Casamassima, Italy; 8 Department of Surgery, Academy of Applied Medical and Social Sciences—AMiSNS, 82-300 Elblag, Poland; l.marano@amisns.edu.pl

**Keywords:** solid pseudopapillary neoplasm of the pancreas, pancreatic neoplasm, pancreas surgery, pancreas

## Abstract

Background: Solid pseudopapillary neoplasms of the pancreas (SPN-P) are rare, low-grade malignancies primarily affecting young women. While surgical resection is definitive, the optimal balance between oncological radicality and functional preservation remains a clinical challenge. This study evaluates tailored surgical strategies utilizing minimally invasive and parenchyma-preserving techniques. Patients and Methods: We conducted a multi-institutional retrospective analysis of SPN-P cases treated between March 2020 and May 2023. Out of 167 pancreatic resections, five paradigmatic cases were identified. We analyzed the decision-making process for preoperative staging (CT/MRI/EUS-FNB), surgical approach (open, laparoscopic, or robotic), and the implementation of parenchyma-preserving versus formal resections. Results: The cohort included four females and one male (mean age 40.6 years; range 13–73). Surgical approaches were tailored to tumor location and patient characteristics: two patients underwent pancreatoduodenectomy (one laparotomic, one laparoscopic), two underwent distal pancreatectomy (one robotic, one laparoscopic), and one pediatric patient underwent laparoscopic parenchyma-preserving central pancreatectomy. R0 resection was achieved in all cases. No Grade B/C postoperative pancreatic fistulas (POPF) or complications Clavien-Dindo ≥III occurred. At a mean follow-up (FU) of 38.4 months (range 20–58), the disease-free survival rate was 100%. One patient developed new-onset diabetes mellitus following distal pancreatectomy. Conclusions: A tailored surgical approach—integrating robotic, laparoscopic, and parenchyma-preserving techniques—may enable excellent oncological outcomes while minimizing morbidity. For SPN-P, the choice of procedure should prioritize the preservation of pancreatic function, particularly in young patients, without compromising surgical margins.

## 1. Introduction

Solid pseudopapillary neoplasm of the pancreas (SPN) is a rare clinical entity, accounting for approximately 0.9–2.7% of all exocrine pancreatic tumors and roughly 5% of cystic pancreatic lesions [1]. Initially characterized by Frantz in 1959, SPN primarily affects young women, with a female-to-male ratio of 9.8:1 and a median age at diagnosis of 28.5 years. Conversely, male patients often present later in life and may exhibit more aggressive biological behaviour and a comparatively poorer prognosis [2,3,4,5].

Despite being classified as a low-grade malignancy, SPN has the potential for local invasion and distant metastasis, most frequently to the liver and peritoneum [1,2]. Clinical presentation is often indolent; many patients remain asymptomatic, with the lesion being discovered incidentally during imaging studies for unrelated concerns. When symptomatic, common manifestations include vague abdominal pain, a palpable epigastric mass, weight loss, or obstructive jaundice. Rarely, acute complications such as spontaneous rupture, gastric outlet obstruction, or portal hypertension may occur [2,3,6,7,8]. Complete surgical resection represents the gold standard of treatment. The choice of procedure—ranging from enucleation (EN) and central pancreatectomy (CP) to radical pancreatoduodenectomy (PD) or distal pancreatosplenectomy (DP)—is dictated by tumor size, anatomical location, and proximity to the main pancreatic duct [2,3]. Given the favourable biology of SPN, parenchyma-preserving resections (PPR) are increasingly favoured to minimize long-term endocrine and exocrine insufficiency. The overall prognosis is excellent, with five-year survival rates exceeding 97% following R0 resection [9].

Managing solid pseudopapillary neoplasms (SPNs) of the pancreas represents a unique surgical challenge. Because these tumors typically affect young patients with a long life expectancy, the goal shifts from simple survival to balancing oncological radicality with the preservation of long-term pancreatic function [10].

However, there are barriers and controversies to an adequate surgical approach to SPNs on a patient-by-patient basis. A “tailored” approach—often involving parenchyma-preserving methods (PPM) like enucleation or central pancreatectomy—faces several hurdles, including the risk of Post Operative Pancreatic Fistula (POPF) parenchyma-preserving surgeries are paradoxically associated with a higher incidence of POPF (approximately 23%) compared to standard resections [10,11]. This risk may deter surgeons from choosing organ-sparing options. Moreover, there are currently no standardized criteria or definitive clinical guidelines for the extent of resection [12]. The lack of unified protocols leads to significant variation in management across different institutions [13].

While SPNs are known to generally have low malignant potential, up to 15–20% can exhibit aggressive behavior, such as local invasion or distant metastasis [12,13]. Current imaging often fails to reliably differentiate indolent cases from those requiring radical oncologic resection.

Also, because SPN is rare, the long-term oncological safety of parenchyma-sparing surgery—with particular regard to local recurrence—remains less documented than standard procedures [10].

This study aims to evaluate our institutional experience with SPN, focusing on tailored surgical decision-making and long-term functional outcomes.

## 2. Patients and Methods

### 2.1. Study Design and Setting

This study is a multi-institutional, retrospective case series conducted in accordance with the Preferred Reporting Of Case Series in Surgery (PROCESS) guidelines. Out of a total pool of 167 pancreatic resections performed between March 2020 and May 2023 at “Policlinico di Bari” University Hospital, Bari, Italy and “G. Panico” Hospital, Tricase, Italy, five consecutive cases of solid pseudopapillary neoplasms (SPN-P) were identified. These cases were selected as “paradigmatic” examples to illustrate the feasibility of tailored surgical decision-making across different anatomical locations and patient age groups.

### 2.2. Patient Selection and Eligibility

Inclusion criteria consisted of the following:

(1) Histologically confirmed SPN-P via endoscopic ultrasound-guided fine-needle biopsy (EUS-FNB) or final surgical pathology;
(2) Primary surgical treatment within the study period;
(3) Available long-term follow-up data.

No patients were excluded due to disease stage or surgical approach.

### 2.3. Preoperative Assessment and Multidisciplinary Review

All patients underwent a standardized diagnostic workup including contrast-enhanced Computed Tomography (CT) and Magnetic Resonance Imaging (MRI) to assess tumor size, location, and vascular involvement. Every case was reviewed by a Multidisciplinary Team (MDT) comprising hepatobiliary surgeons, radiologists, oncologists, and pathologists (with the addition of pediatricians for pediatric cases) to determine the optimal surgical strategy.

### 2.4. Surgical Intervention and Tailoring

Surgical procedures were performed by senior consultant surgeons with expertise in minimally invasive and parenchyma-preserving pancreatic surgery. The choice of approach (open, laparoscopic, or robotic) and procedure (formal resection vs. parenchyma-preserving techniques like central pancreatectomy) was tailored to the tumor’s anatomical relationship with the main pancreatic duct and major vessels, as well as the patient’s functional needs.

### 2.5. Outcomes and Data Collection

We analysed the decision-making process for preoperative staging (CT/MRI/EUS-FNB), surgical approach (open, laparoscopic, or robotic), and the implementation of PPR versus formal resections.

The primary outcome was the feasibility of the tailored surgical strategy.

Secondary outcomes included the following:

- Overall perioperative complication rate;
- The rate of postoperative pancreatic fistula defined and graded by International Study Group for Pancreatic Surgery (ISGPS) criteria, through measurement of amylase levels in drainage fluid at postoperative day (POD) 1, 3 and 5;
- Status of surgical margin (R0/R1);
- Long-term disease-free survival;
- POD of resumption of oral intake;
- POD of resumption of bowel function;
- POD of drain removal.

To this purpose, we collected patients’ demographics and disease data, results of the preoperative blood tests, imaging features of the neoplasm (size in cm, location in the pancreas) as documented on CT or MRI scans, postoperative pancreatic fistula rate and pathology including margin status.

Data were extracted from a prospective, deidentified database and verified against electronic medical records by two independent researchers.

### 2.6. Follow-Up Protocol

The postoperative surveillance included metabolic monitoring (blood glucose and HbA1c), nutritional assessment, and oncological imaging (CT or MRI) with serum tumor markers (Ca 19-9), performed every 3–6 months for the first two years and annually thereafter.

## 3. Results

All patients were reviewed at follow-up. Mean follow-up duration was 38.4 months.

### 3.1. Case 1

A 73-year-old man presented with progressive jaundice and abdominal pain for about a week. His medical history was significant only for arterial hypertension. Contrast-enhanced thoracic and abdominal CT and MRI revealed a vascularized solid lesion with cystic areas, suggestive of a SPN in the pancreatic head, measuring approximately 4 × 3 × 5 cm. Blood tests and tumor markers were unremarkable.

After a multidisciplinary discussion (MDD), the patient underwent an open pylorus-preserving PD, which included an end-to-side pancreatojejunostomy, an end-to-side hepaticojejunostomy, and an end-to-side duodenojejunostomy. The post-operative period was uneventful. The patient mobilized on postoperative day (POD) 1, resumed oral intake on POD 4, and regained bowel function by POD 3. Abdominal drains were removed on POD 6 after confirming negative amylase levels in the drainage fluid on PODs 1, 3, and 5. The patient was discharged on POD 10. Histopathological analysis confirmed the diagnosis of SPN-P, staged as pT3 R0 according to the UICC 2017 criteria [14]. Oncologists recommended clinical surveillance with abdominal MRI at 4 months, then every 6 months for 2 years, and annual FU for a total of 5 years. Approximately five years post-surgery, the patient remains disease-free, with no evidence of diabetes, exocrine insufficiency, or recurrence.

### 3.2. Case 2

A 45-year-old woman presented with diffuse abdominal pain persisting for approximately three months. Her clinical history was notable only for gastroesophageal reflux disease (GERD). Imaging studies, including a contrast-enhanced abdominal CT scan and MRI, revealed a solid lesion in the pancreatic body measuring approximately 3 × 2.5 × 3 cm. The lesion exhibited well-defined margins and no evidence of local infiltration, while the Wirsung duct was regular. Blood tests and tumor markers were unremarkable. EUS-FNB provided a histological diagnosis of SPN-P.

After MDD, the patient underwent a robotic DP. The postoperative course was uneventful. The patient mobilized and resumed oral intake on POD 1, regained bowel function by POD 3, and had abdominal drains removed on POD 4 after confirming negative amylase levels in the drainage fluid on PODs 1 and 3. The patient was discharged on POD 6 following a postoperative abdominal CT scan, which showed normal surgical findings. Histopathological analysis confirmed the diagnosis of SPN-P, staged as pT2 R0 according to the UICC 2017 criteria [14]. A clinical surveillance strategy was recommended, consisting of abdominal MRI or CT at 4 months, then every 6 months for two years, followed by annual FU for five years. During nutritional FU a few months after surgery, the patient developed glycemic abnormalities, requiring initiation of antidiabetic therapy. After approximately five years of oncological FU, there are no signs of clinical recurrence.

### 3.3. Case 3

A 48-year-old woman presented with persistent epigastric pain lasting several months. Her medical history was significant for GERD, a hiatal hernia, and a prior right knee surgery. Blood tests, including tumor markers, were within normal limits. Esophagogastroduodenoscopy (EGD) confirmed the presence of a hiatal hernia but revealed no solid gastric lesions. Further imaging, including contrast-enhanced abdominal CT and MRI, identified a 6 × 4 × 5 cm round lesion in the pancreatic head. The lesion exhibited vascularized solid components intermixed with cystic areas, suggestive of a SPN. The main bile duct and Wirsung duct were not dilated, and there was no evidence of local infiltration. EUS-FNB was performed but was not diagnostic.

Considering the patient’s persistent symptoms, lesion size, and radiological findings, the multidisciplinary team recommended upfront surgical intervention. The patient subsequently underwent a laparoscopic Whipple procedure, which included an end-to-end pancreatojejunostomy, an end-to-side hepaticojejunostomy, and an end-to-side gastrojejunostomy. The postoperative course was uneventful. The patient mobilized early and initiated oral intake on POD 1, regained bowel function by POD 2, and had negative amylase levels in the drainage fluid on PODs 1, 3, and 5. Abdominal drains were removed on POD 7 following a confirmatory abdominal CT scan. The patient was discharged on POD 8. Histopathological examination confirmed the diagnosis of SPN-P, staged as pT3 N0 R0 according to UICC 2017 criteria [14]. Peripancreatic lymphadenectomy was performed, yielding 24 lymph nodes, all negative for malignancy. Oncological FU recommendations included clinical surveillance with abdominal MRI or CT at 4 months, every 6 months for 2 years, and annual FU for a total of 5 years. Approximately three years postoperatively, the patient remains asymptomatic, with no evidence of clinical recurrence or signs of exocrine or endocrine pancreatic insufficiency.

### 3.4. Case 4

**Figure 1:**
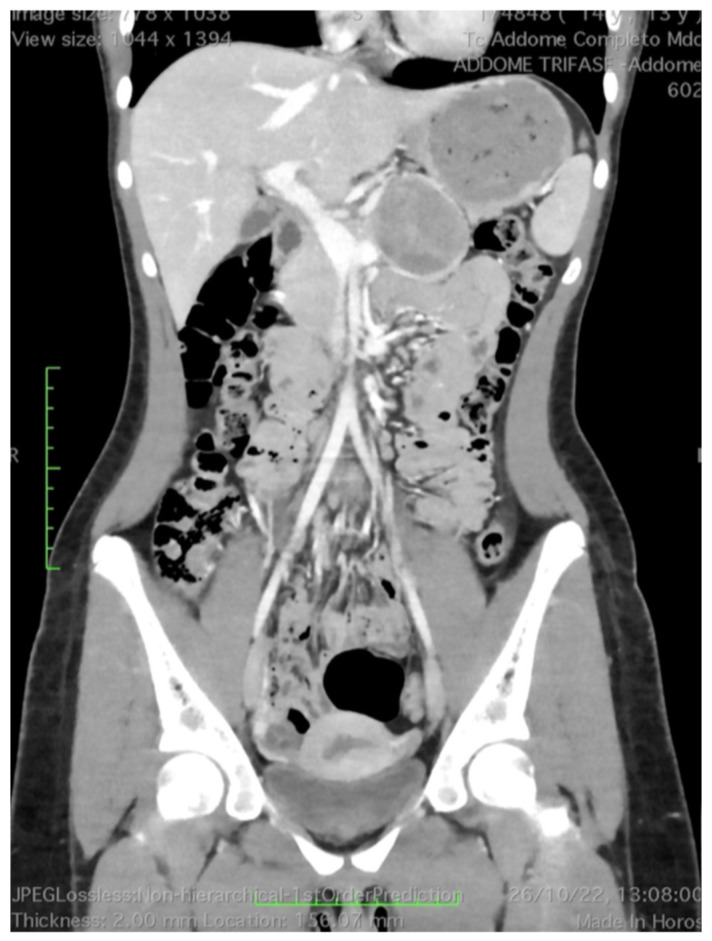
Coronal contrast-enhanced CT scan of the abdomen (portal venous phase). A large, well-defined heterogeneous mass is located at the superior margin of the pancreatic neck and body, measuring approximately 5 × 4 × 5.5 cm. The lesion displays smooth borders and a mixed composition consisting of hypodense cystic areas and hyperdense solid components. The mass is positioned cranially to the splenic vein, which shows luminal narrowing due to extrinsic compression, and inferiorly to the splenic artery, which is displaced cranially. No signs of loss of the fat plane or irregular vessel wall contouring are observed. Green scales are measures of length indicated in cm.

A 13-year-old girl presented to the emergency department with a one-week history of abdominal pain localized to the lower quadrants, unresponsive to analgesic therapy. She reported a history of irregular periods, frequently associated with dysmenorrhea that was poorly responsive to nonsteroidal anti-inflammatory drugs (NSAIDs). The patient was admitted to the Pediatric Unit with the suspicion of gynecological pathology. Blood tests, including tumor markers, were within normal limits. Abdominal ultrasonography (US) revealed an isoechoic, round lesion measuring approximately 4 cm, located between the tail of the pancreas and the posterior wall of the stomach. Further imaging studies, including contrast-enhanced CT and MRI of the abdomen, confirmed a lesion at the superior margin of the pancreatic neck-body region, measuring approximately 5 × 4 × 5.5 cm. The lesion exhibited smooth, well-defined margins and a mixed composition, characterized by a cystic component with dense material, microcalcifications, and intralesional solid component demonstrating post-contrast enhancement, particularly during the arterial phase. Imaging also indicated compression of the splenic vein, which remained patent, and cranial displacement of the splenic artery without evidence of vascular infiltration (Figure 1). EUS-FNB provided a definitive diagnosis of a SPN-P.

**Figure 2:**
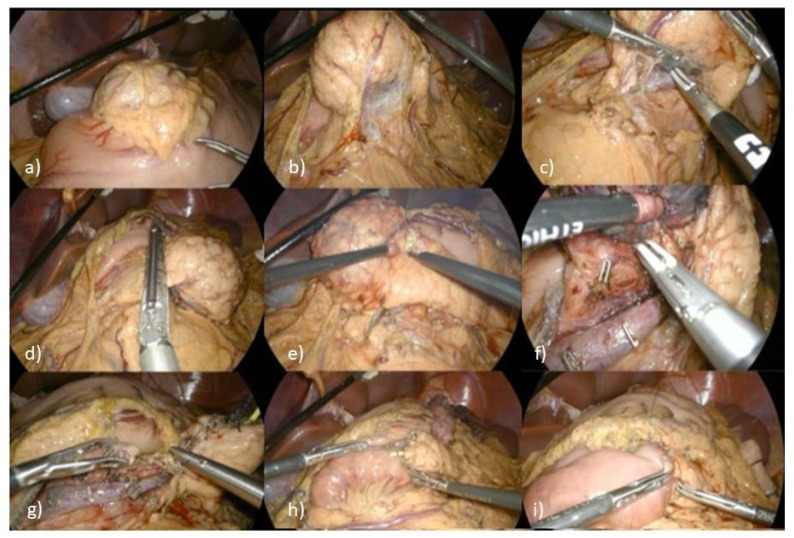
Intraoperative steps of laparoscopic central pancreatectomy. (**a**,**b**) Laparoscopic view showing a voluminous, well-defined mass with smooth margins located at the superior border of the pancreatic neck-body region. (**c**,**d**) Dissection and mobilization of the lesion from the retroperitoneal space, demonstrating the relationship between the mass and the splenic vessels. (**e**,**f**) Transection of the pancreatic parenchyma at the proximal (head-neck) and distal (body-tail) margins of the lesion. (**g**,**h**) Isolation of the distal pancreatic remnant (tail) and preparation of a Roux-en-Y jejunal loop for reconstruction. (**i**) Final view of the end-to-end pancreatojejunostomy, showing the anastomosis between the distal pancreatic tail and the jejunal limb.

The case was reviewed by a multidisciplinary team comprising pediatricians, surgeons, radiologists, oncologists, and pathologists, who recommended surgical management (Figure 2). The patient underwent laparoscopic CP with an end-to-end pancreatojejunostomy in a defunctionalized Roux-en-Y loop.

**Figure 3:**
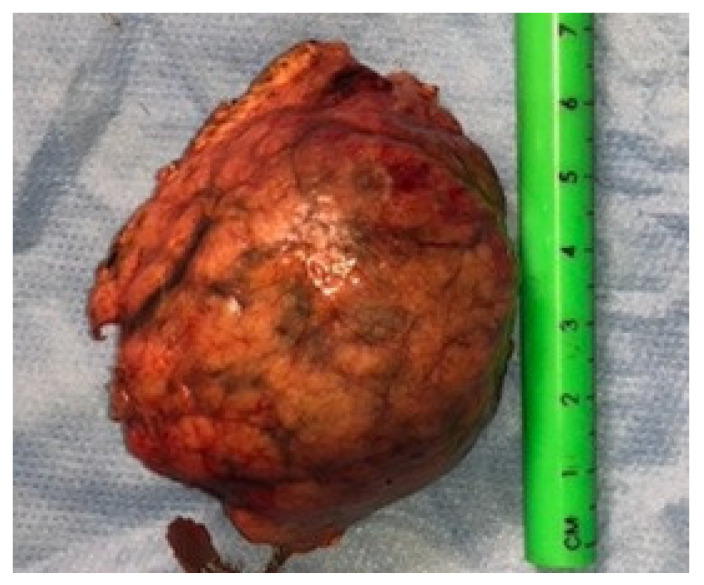
Resected SPN specimen after minimally invasive central pancreatectomy.

The postoperative course was uneventful, with early mobilization and reintroduction of oral intake on POD1. Bowel function resumed on PODs 3–4. Abdominal drains were removed on POD 8 after confirming normal findings on a postoperative CT scan and negative amylase levels in drainage fluid on PODs 1, 3, and 5. The patient was discharged on POD 8. Histopathological examination confirmed the diagnosis of SPN-P, staged as pT3 R0 according to UICC 2017 criteria (Figure 3) [14]. A single peri-lesional lymph node was isolated and found to be negative for malignancy. Oncologists recommended clinical surveillance, including abdominal MRI at 4 months, every 6 months for 2 years, and annual FU for 5 years. The patient continues to be monitored by a specialist in oncological nutrition. Approximately two years post-surgery, the patient remains disease-free, with no evidence of diabetes, exocrine insufficiency, or recurrence.

### 3.5. Case 5

A 24-year-old woman with a two-year history of dyspeptic symptoms underwent abdominal US, which revealed a pancreatic mass. Subsequent imaging studies, including contrast-enhanced CT and MRI of the abdomen, confirmed a lesion in the pancreatic tail measuring approximately 3.6 × 2.7 × 3 cm. The lesion had well-defined margins, a heterogeneous contrast enhancement pattern, and peripheral hemorrhagic areas. Blood tests, including tumor markers, were within normal limits. EUS was performed, confirming the diagnosis of SPN-P.

Following an MDD, the patient underwent a laparoscopic DP. The postoperative course was uneventful. The patient demonstrated early mobilization and tolerance to oral intake by POD 1, with bowel function resuming on PODs 2–3. Amylase levels in drainage fluid were negative on PODs 1–3, prompting the removal of abdominal drains. The patient was discharged on POD 4. Histopathological examination confirmed the diagnosis of SPN-P, with no evidence of malignancy in six examined loco-regional lymph nodes (pT2 N0 R0, according to UICC 2017 criteria) [14]. Oncologists recommended a postoperative surveillance protocol consisting of abdominal MRI at 4 months, followed by imaging every 6 months for 2 years, and annual FU for 5 years. Approximately two year post-surgery, the patient remains asymptomatic, with no evidence of clinical recurrence or pancreatic insufficiency.

## 4. Discussion

SPN-P is an uncommon pancreatic neoplasm classified under the 2019 WHO classification with a strong predilection for young females [1]. Advances in imaging and increased awareness of this pancreatic neoplasm have improved detection rates [3]. Although specific risk factors remain unidentified, association with familial adenomatous polyposis have been reported [15,16]. The etiology and histogenesis of SPN-P remain unclear. Cytological analyses suggest no correlation with any specific cell lineage. Recent evidence posits a non-pancreatic origin, suggesting derivation from pluripotent genital ridge cells that migrate to the pancreas during embryogenesis. Genetic and morphological similarities between ovarian and pancreatic SPN cells support this hypothesis [17,18].

From March 2020 to May 2023, four women and one man with a mean age of 40.6 years (range: 13–73) were diagnosed with SPN-P and underwent surgical treatment at our Institutions (Table 1). As reported in literature, the most frequent symptoms were abdominal pain and dyspepsia [2,3]. Most patients are asymptomatic at diagnosis. They may occasionally present with a gradually enlarging abdominal mass or vague abdominal pain. All patients presented with normal blood tests and negative tumor markers, and underwent a comprehensive diagnostic workup, including contrast-enhanced abdominal CT and MRI. SPN-P frequently contain varying amounts of necrosis, hemorrhage, and cystic change. Lesions can be large at the time of diagnosis (median size ~8 cm) [19,20]. Elevated β-HCG has been reported [21].

Preoperative imaging is the most widely used tool for diagnosing SPN. On ultrasound, SPNs are large, well-defined masses with heterogeneous appearances, due to their solid and cystic composition. On CT scan, typical SPTs are larger than 3 cm and are well-defined lesions with central cystic and peripheral solid components. 

Contrast enhancement may be heterogeneous due to hemorrhagic degeneration. Small atypical tumors are well marginated and solid with poor pancreatic phase enhancement, slowly progressing. Large atypical SPTs may be partly calcified solid masses or cystic masses [22]. MRI typically demonstrates a well-defined lesion. It may show a pure solid consistency in ~80% of cases [23]. Reported signal characteristics include low to heterogeneous signal intensity in T1, heterogeneous to high signal intensity in T2, and early heterogeneous and slowly progressive enhancement in gadolinium-contrast T1 sequences [23,24].

Differential diagnosis should be considered with pancreatic neuroendocrine tumors: they can have similar imaging features and be difficult to differentiate on imaging [25]. Diagnosis of SPN requires the expertise of experienced specialists, as it has been noted that up to one third of images may not be typical [26].

In our cohort, two patients underwent surgery without a pre-operative biopsy due to the evident radiological diagnosis, while histological confirmation of SPN was obtained preoperatively for the other patients. Following MDD, all patients were deemed suitable candidates for surgical resection.

While SPN-P is considered a low-grade malignancy, surgical resection remains the cornerstone of treatment, with the approach depending on tumor location and size [20]. SPNs are often found incidentally, and because of their low-grade nature, they are rarely fatal if resected early. PPR are preferred for smaller tumors, with options including enucleation, central pancreatectomy, or spleen-preserving distal pancreatectomy [27]. Major resections typically refer to PD for head tumors or DP for tail tumors [28]. A minimally invasive, laparoscopic or robotic approach is often used to reduce hospital stay and complications, in the hands of experienced surgeons with proficiency in minimally invasive pancreatic resections [29]. A “tailored” approach must balance oncological radicality with the preservation of long-term quality of life. In our series, we utilized a spectrum of surgical techniques: Radical Resection: PD (Cases 1 and 3) was reserved for large lesions in the pancreatic head where preservation was anatomically unfeasible; PPR: CP (Case 4) was successfully employed in a pediatric patient to prevent the lifelong metabolic consequences of a major resection; Minimally Invasive Surgery: The use of robotic (Case 2) and laparoscopic (Cases 3, 4, 5) platforms facilitated rapid recovery, with a mean discharge of 7.4 days. In recent years, the incidence of PPR has increased, which entails preservation of endocrine and exocrine function [30]. While not observed in our cohort, the management of advanced SPN requires a multimodality approach. In locally advanced SPNs, aggressive surgery is justified, as the disease is often curable even with invasion into adjacent organs [31]. Locally advanced cases may necessitate en bloc resection of the pancreas along with involved adjacent structures to achieve complete tumor removal. While routine lymphadenectomy has been shown to be unnecessary, suspicious lymphadenopathy should be carefully evaluated and removed during surgery [32]. For resectable liver metastases, synchronous resection is recommended, as it often provides a survival benefit. Liver metastases, when resectable, may be managed with synchronous surgical resection. Despite the absence of specific recommendations for patients with unresectable liver metastatic lesions, cases of selective internal radiotherapy with Yttrium-90 [33], chemoembolization with percutaneous hepatic perfusion of Melphalan [34], combination of tyrosine kinase inhibitor Sunitinib and hepatic artery embolization [35], radiofrequency ablation, and liver transplantation [36] are described in the literature. On the other hand, in the case of peritoneal metastases, these can be subjected to complete cytoreductive surgery and intraperitoneal chemo hyperthermia (HIPEC) with Irinotecan and Oxaliplatin [37]. Finally, the treatment of unresectable SPNs-P with adjuvant chemotherapy has been described, but there is no recommendation [36,38,39]. Generally, chemotherapy has a limited role, though it may be considered for unresectable, highly advanced tumors. Following resection, considering its low-grade malignancy, the management of the pancreatic stump remains a critical issue in order to minimize the incidence of post-operative complications and, above all, of POPF [40]. The post-operative course was uneventful in all cases, with no instances of POPF, intra-abdominal abscess, wound infection, or systemic infection (Table 1). Strategies to reduce the incidence of POPF continue to evolve. Techniques such as the Blumgart anastomosis and modifications in pancreaticogastrostomy have been explored to improve post-operative outcomes and reduce morbidity following pancreatic resections [41]. Additionally, ensuring that management and outcome standards remain within established benchmarks is essential. Benchmarking in pancreatic surgery plays a crucial role in assessing surgical outcomes and optimizing patient care. The use of standardized metrics for pancreatic resections allows for comparisons across institutions, ultimately guiding improvements in surgical techniques and perioperative management [42].

Long-term FU is necessary due to the possibility of recurrence (roughly 2–14% of cases). Patients in our series were followed up for a minimum of 20 months, with a mean follow-up duration of 38.4 months (range: 20–58 months). FU was conducted in collaboration with oncologists and specialized oncological nutritionists. No cases of clinical or radiological recurrence were observed, and all patients remain alive at the time of reporting. The only post-operative complication observed was the onset of diabetes mellitus in one patient who had undergone DP for an SPN-P located in the pancreatic body, highlighting the metabolic consequences of pancreatic resections.

The prognosis for SPN-P is favorable, with a reported 5-year survival rate of approximately 97% following complete surgical resection [9]. A comprehensive review in 2014 reported a mortality rate of only 1.5% among 1952 SPN-P patients [3]. Recurrence rates range from 2% to 10%, with poor prognostic factors including male sex, positive surgical margins (R1), lymph node involvement, perineural or vascular invasion, adjacent structure infiltration, high proliferation index, and undifferentiated carcinoma [43,44,45]. Our findings align with the existing literature, further reinforcing the excellent prognosis associated with complete surgical resection of SPN-P. Notably, the development of diabetes mellitus post-DP in one of our patients underscores the importance of pancreatic function preservation when feasible, particularly in younger patients [26].

The primary limitation of this study is the small sample size and its retrospective, single-center design, which may introduce inherent selection bias. As a descriptive case series of five consecutive patients, the results lack a control group or benchmarking, preventing the formulation of generalized statistical conclusions or definitive evidence of superiority for any specific surgical technique. However, these cases were selected as paradigmatic examples to illustrate the feasibility of a tailored, multidisciplinary approach to varied clinical presentations of SPN-P.

In rare tumors, large-scale randomized controlled trials are often impossible. Evidence from this series, albeit being a small sample, may help in bridging the knowledge gap in p-SPNs, eventually forming the basis for meta-analyses and systematic reviews [11].

With this study, we demonstrate the technical feasibility of p-SPN resection through different surgical approaches taken to reach adequate treatment, providing a technical roadmap for other surgeons facing similar unique anatomical or clinical challenges. By reporting on varied treatment corridors, we also highlight the need for specialized experience, as centralizing care for these complex, rare tumors has been advocated to improve global outcomes by concentrating expertise [13].

A tailored surgical approach is a direct application of precision medicine in the surgical theatre. We demonstrated an approach towards individualized risk assessment, moving away from “one-size-fits-all” radical surgery. Patient-specific factors (age, tumor location, and potential genetic markers like the CTNNB1 mutation) may be used to determine the surgical margin [13,18]. In the context of precision oncology, this study also shows how quality of life was used as a metric, with the success of a treatment depending not just on tumor removal but also on the avoidance of long-term morbidities like endocrine (diabetes) and exocrine insufficiency [10], though more factors are likely involved. Emerging research suggests that SPNs may have different prevalences or behaviors across different populations (e.g., higher frequency in certain Asian populations), requiring population-specific precision strategies [13].

While minimally invasive approaches, including robotic and laparoscopic techniques, offer promising outcomes, further studies with larger cohorts are needed to refine optimal surgical strategies, assess long-term metabolic impacts, and explore strategies for preserving pancreatic endocrine function in SPN-P patients.

## 5. Conclusions

Surgical resection may enable excellent outcomes for patients with SPN-P. Parenchyma-preserving techniques, when oncologically safe, may represent an option to minimize pancreatic insufficiency. An individualized approach may benefit young patients with a view to minimize lifelong metabolic morbidities, such as new-onset diabetes. Long-term surveillance remains essential due to the potential for late recurrence.

## Figures and Tables

**Table 1:** Comparison of Clinical and Pathological Data.

Case	Age/Sex	Location	Surgery	Approach	Perioperative Complications	Stage (UICC)	Follow-Up (mo)	Status
1	73/M	Head	PPPD	Open	None	pT3 R0	58	NED
2	45/F	Body	DP	Robotic	None	pT2 R0	54	NED (DM+)
3	48/F	Head	PD	Laparoscopic	None	pT3 N0 R0	36	NED
4	13/F	Neck/Body	CP	Laparoscopic	None	pT3 R0	24	NED
5	24/F	Tail	DP	Laparoscopic	None	pT2 N0 R0	20	NED

Legend: NED—No Evidence of Disease; DM—Diabetes Mellitus; PPPD—Pylorus Preserving Pancreatico—Duodenectomy; DP—Distal Pancreatectomy; PD—Pancreaticoduodenectomy; CP—Central Pancreatectomy.

## Data Availability

The data that support the findings of this study are available from the corresponding author upon reasonable request.
